# Determinants of stunting in Indonesian children: evidence from a cross-sectional survey indicate a prominent role for the water, sanitation and hygiene sector in stunting reduction

**DOI:** 10.1186/s12889-016-3339-8

**Published:** 2016-07-29

**Authors:** Harriet Torlesse, Aidan Anthony Cronin, Susy Katikana Sebayang, Robin Nandy

**Affiliations:** 1 UNICEF Indonesia, World Trade Center 6 (10th Floor), Jalan Jenderal Sudirman Kav. 31, Jakarta, 12920 Indonesia; 2 Faculty of Public Health, University of Airlangga, Banyuwangi Campus, Jalan Wijaya Kusuma No 113, Kecamatan Giri, Banyuwangi Indonesia

**Keywords:** Indonesia, Stunting, Sanitation, Household water treatment

## Abstract

**Background:**

Stunting in early life has considerable human and economic costs. The purpose of the study was to identify factors associated with stunting among children aged 0-23 months in Indonesia to inform the design of appropriate policy and programme responses.

**Methods:**

Determinants of child stunting, including severe stunting, were examined in three districts in Indonesia using data from a cross-sectional survey conducted in 2011. A total of 1366 children were included. The analysis used multiple logistic regression to determine unadjusted and adjusted odds ratios.

**Results:**

The prevalence of stunting and severe stunting was 28.4 % and 6.7 %, respectively. The multivariate analysis on determinants of stunting identified a significant interaction between household sanitary facility and household water treatment (*P* for interaction = 0.007) after controlling for potential covariates: in households that drank untreated water, the adjusted odds on child stunting was over three times higher if the household used a unimproved latrine (adjusted odds ratio 3.47, 95 % confidence interval 1.73-7.28, *P* <0.001); however, in households that drank treated water, the adjusted odds on child stunting was not significantly higher if the household used an unimproved latrine (adjusted odds ratio 1.27, 95 % confidence interval 0.99-1.63, *P* = 0.06). Other significant risk factors included male sex, older child age and lower wealth quintile. The risk factors for severe stunting included male sex, older child age, lower wealth quintile, no antenatal care in a health facility, and mother’s participation in decisions on what food was cooked in the household.

**Conclusions:**

The combination of unimproved latrines and untreated drinking water was associated with an increased odds on stunting in Indonesia compared with improved conditions. Policies and programmes to address child stunting in Indonesia must consider water, sanitation and hygiene interventions. Operational research is needed to determine how best to converge and integrate water, sanitation and hygiene interventions into a broader multisectoral approach to reduce stunting in Indonesia.

**Electronic supplementary material:**

The online version of this article (doi:10.1186/s12889-016-3339-8) contains supplementary material, which is available to authorized users.

## Background

Failure to grow and develop optimally in early life has considerable human and economic costs [1]. Stunting increases the risk of child deaths, adversely affects cognitive and motor development, lowers performance at school, increases the risk of overnutrition and non-communicable diseases, and reduces productivity in adulthood [2]. These accumulative effects cost African and Asian countries up to 11 % of their gross national product [3].

Stunting, or low height for age, is caused by long-term insufficient nutrient intake and/or frequent infections. Despite Indonesia’s middle income status, the country carries the fifth highest burden of stunted children in the world [4]. There has been negligible change in the stunting prevalence in the last decade, and if current trends continue, the country is unlikely to achieve the 2012 World Health Assembly goal to reduce stunting by 40 % by 2025 [1]. Over one-third (37 %) of children aged less than five years were stunted in 2013 and the prevalence exceeded 40 % in 15 out of 33 provinces; 18 % of children were severely stunted [5].

Indonesia joined the global Scaling Up Nutrition (SUN) Movement in 2011 and launched its national movement in 2013 to galvanize action across multiple sectors to reduce stunting and other forms of undernutrition. Both the widely accepted conceptual framework for optimal nutrition [2] and the policy framework for the SUN Movement in Indonesia [6] recognize the need for a multisector actions. However, information on the determinants on stunting in Indonesia to inform the design of multi-sector programmes is lacking, and the operationalization of multisector response is yet to be realised [7].

**Fig. 1:**
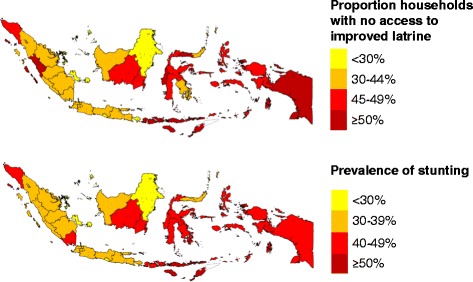
Proportion of households without access to an improved latrine and prevalence of stunting in child stunting. Provincial estimates of the proportion of households without access to an improved latrine and the prevalence of stunting in children aged 0-59 months in Indonesia (derived from [5])

There is close symmetry between the provincial-level maps for sanitation and child stunting in Indonesia (Fig. 1). Using data collected by the 2013 Basic Health Research Survey [5], we determined that provincial estimates of the proportion of households with access to an improved latrine are inversely correlated with provincial estimates of the percentage of stunted children aged less than five years (R^2^ = 65.7 %, *P* < 0.001). To examine the relationship further, this study utilized data from a cross-sectional survey in three districts in Indonesia to examine maternal, child and household determinants of stunting and severe stunting in children, including indicators on household water, sanitary and hygiene (WASH) facilities and practices.

## Methods

### Subjects

The analysis used data from a baseline survey conducted in three districts in Indonesia for the European Union funded Maternal and Young Child Nutrition Security Initiative (MYCNSIA) project between November and December 2011. The MYCNSIA project was designed to reduce stunting in children aged less than three years and anaemia in pregnant women through the scale-up of nutrition-specific and nutrition-sensitive interventions.

The three MYCNSIA focus districts were selected to represent three different typologies in Indonesia: Sikka is a coastal district in East Nusa Tenggara Province that has one of the highest stunting prevalence figures in the country; Jayawijaya is a remote highland district in Papua Province where many social and health indictors are well below the national average; and Klaten is a densely populated district in Central Java where the burden of stunting is high.

Households were eligible for inclusion in the survey if they contained a child aged 0-35 months and if the primary caregiver was present for interview. Children were excluded if a household member reported that they had a chronic disease lasting more than three months such as cerebral palsy, asthma or diabetes.

### Sampling design

The survey used a multistage cluster sampling design to produce statistically representative data for the three districts as a single sample universe. A desired sample size of 2009 children aged 0-35 months was calculated to detect a 5 percentage point decline in the estimated baseline stunting prevalence of 46.4 % using a one-tail test at a power of 80 %, confidence level of 95 %, design effect of 1.5 and with 10 % additional children to allow for missing data. A total of 102 clusters, comprising census blocks created by the Indonesia Central Bureau of Statistics, were selected from the three districts using probability proportionate to size sampling (70 clusters in Klaten, 19 in Sikka and 13 in Jayawijaya). In each cluster, 20 households were selected using simple random sampling from a list of households with children aged 0-35 months in each census block. If the cluster did not have 20 eligible households, a contiguous cluster was visited to randomly select the missing number of households; the final number of census blocks was 193 (148 in Klaten, 25 in Sikka and 20 in Jayawijaya). If a household had more than one child aged 0-35 months, only the youngest child was included.

### Data collection

A structured pre-tested questionnaire was used to record data on children aged 0-35 months, their mothers, and their households. The data included the child’s age, sex, weight and height/length; mother’s age, level of education and participation in household decisions; breastfeeding, complementary feeding and handwashing practices; child’s access to health and nutrition services (growth monitoring, micronutrient supplementation, and immunization); mother’s access to health services (antenatal care, assistance at delivery and place of delivery); household water, sanitation and hygiene; and socio-economic characteristics of the household.

Child weight was measured using SECA electronic scales to a precision of 0.1 kg and child length (for children aged 0-23 months) or height (for children aged 24-35 months) was measured using a locally made height/length board to a precision of 0.1 cm. The SECA weighing scales were calibrated every morning, prior to data collection, using a 5 kg standard weight. Duplicate measurements of anthropometry were performed for 10 % of samples; the within-subject coefficient of variation from the duplicate measurements in both children and women was less than 5 %. All enumerators received at least two days training prior to data collection, and those responsible for taking anthropometric measurements received an additional day of training. Supervisors were assigned to oversee the work of the enumerators and facilitate good rapport with community members.

### Data entry and analysis

Data entry operators entered the data using EPI Info version 7.0.8.0 (Centre for Disease Control, Atlanta, Georgia, USA). Approximately 10 % of the questionnaires were entered in duplicate and keystroke errors were found in less than 0.7 % of fields. A series of checks were performed for duplicate entries, outliers and missing data.

Data analysis was conducted using Stata 11.0 (StataCorp, College Station, Texas, USA). Data were adjusted for cluster sampling using the Generalized Estimating Equation model [8] and robust estimates with xtgee command in Stata 11.0. The analysis focused on children aged 0-23 months because data on infant and young child feeding (IYCF) practices and access of mother to antenatal and delivery care services were not available for children aged 24-35 months and these indicators were potential determinants of stunting. The analysis was repeated for children aged 0-35 months with the caveat that indicators on IYCF practices and maternal health care were not included. Children with missing or invalid data for any of the variables examined were not included in the analytical sample.

Stunting and severe stunting were defined as the proportion of children whose weight-for-height z-score was below -2 standard deviations and -3 standard deviations, respectively, of the median height-for-age of the World Health Organization (WHO) Child Growth Standards [9].

WHO definitions for indicators of IYCF practices in children aged less than two years were applied [10]. In addition, we constructed a variable on “age-appropriate feeding” that combined exclusive breastfeeding for children aged 0-5 months with minimum acceptable diet for children aged 6-23 month; a child was considered to have age-appropriate feeding if she/he was aged 0-5 months and exclusively breastfed or aged 6-23 months and consumed a minimal acceptable diet.

An index of household wealth was constructed using data on household assets (materials used to construct the floor, walls and roof of the house, fuel for cooking, water source, sanitation facility, and ownership of livestock, vehicles and electronic goods) and five quintile categories were created.

The operational definitions used by the UNICEF and WHO’s Joint Monitoring Programme for improved drinking water supply, treated water, and improved sanitation facilities were applied [11]. The household water source is considered “improved” if it was piped into the dwelling, yard or plot, a public tap, a protected well in the dwelling, yard or plot, a protected public well, or rainwater. The household water is considered “treated” if the household reported that it was boiled, bleached, filtered or solar disinfected. The household sanitary facility is considered “improved” only if it was a latrine with a septic tank. The disposal of a child’s faeces was considered safe only if it was discarded in an improved latrine.

In models using stunting or severe stunting as the dependent variables, we report on unadjusted odds ratios (OR) and adjusted odds ratios (AOR) and 95 % confidence intervals (CI) for variables. The selection of variables was based on the conceptual framework for optimal nutrition [2]. Multiple logistic regression included variables that had a *P*-value <0.25 in the univariate analysis. Variables were analysed using the backward elimination and those with a *P*-value <0.05 in multivariate analysis were retained in the final logistic regression model. For all tests, a *P*-value < 0.05 was considered statistically significant. Interactions were considered significant for *P* values <0.01.

## Results

The survey included a representative sample of 2023 children aged 0-35 months, including 1424 children aged 0-23 months. Unless otherwise stated, the analysis presented here relates to 1366 (95.9 %) children aged 0-23 months whose mother was available for interview and who had valid data for all variables examined. Valid stunting data was missing for 3 children. Information on the characteristics of the mother, child or household were unavailable for a further 55 children, either because the mother was not available at the time of interview or the mother was unable to provide this information. Of these 55 children, 13 (23.6 %) were stunted.

### Characteristics of the sample

Table 1 provides data on the socio-economic characteristics of the mothers of children aged 0-23 months and their households. Four percent of mothers were aged less than 20 years, and 14.4 % of mothers did not complete primary education. Less than two-thirds of households (61.1 %) had access to an improved latrine, and 43.4 % practiced safe disposal of children’s faeces. Only 55.3 % of households reported that they used soap for handwashing. Less than one third (31.6 %) of households had an improved source of drinking water, however, 90.0 % of households reported that they treated their water before drinking. Coverage of antenatal care (ANC) by mothers of the children during their most recent pregnancy was high: around 95 % of mothers accessed ANC at least four times during pregnancy, received ANC from a doctor or midwife, and in a private or public health facility. Mothers’ participation in household decisions was also high: 88.7 % mothers participated in household decisions on food, 89.5 % on what food to cook for the household; 95.2 % on what food was given to the child; and 86.4 % on seeking health care for the child.

**Table 1:** Socio-economic characteristics of the population (*N* = 1366)

	Proportion (%)
Mother’s age	
< 20 years	4.0
20-29 years	51.5
30-39 years	37.8
≥ 40 years	6.7
Mothers’ education	
No or incomplete primary education	14.4
Completed primary education	16.8
Completed junior high education	24.4
Completed senior high education	44.5
Number of household members	
≤ 4 people	40.3
> 4 people	59.7
Household water, sanitation and hygiene	
Improved sanitary facility	61.1
Safe disposal of child’s faeces	43.4
Use of soap for handwashing	55.3
Improved source of drinking water	31.6
Treated water	90.0
Mother’s care during last pregnancy and delivery	
At least 4 antenatal care visits	93.3
Antenatal care provided by doctor/midwife	95.3
Antenatal care in a private or public health facility	96.5
Mother participates in household decisions	
Household purchases on food	88.7
What food to cook for the household	89.5
What food to give to child	95.2
Seeking health care for child	86.4
Wealth quintile	
Lowest	21.5
Second	20.3
Third	20.0
Fourth	18.7
Highest	19.5

Table 2 provides data on the nutritional status of the children and infant and young child feeding practices. The prevalence of total stunting (moderate and severe) was 28.4 % and the prevalence of severe stunting was 6.7 %. About two-thirds (67.9 %) of children were breastfed within one hour of birth, and 54.1 % of children aged less than six months were exclusively breastfed. Only 36.6 % of children aged 6-23 months were fed a minimum acceptable diet comprising adequate number of milk feeds, meals and food groups. Overall, 40.6 % of children aged 0-23 months had age-appropriate feeding, defined here as exclusive breastfeeding for children aged 0-5 months and minimum acceptable diet for children aged 6-23 months.

**Table 2:** Background characteristics of children aged 0-23 months (*N* = 1366 unless otherwise stated)

	Proportion (%)
Sex	
Girl	49.6
Boy	50.4
Age	
0-5 months	22.6
6-11 months	25.0
12-23 months	52.4
Stunting	
Moderate stunting	21.7
Severe stunting	6.7
Total stunting	28.4
IYCF practices	
Breastfeeding initiated within one hour of birth (children 0-23 months)	67.9
Exclusive breastfeeding (children 0-5 months)^a^	54.1
Minimum dietary diversity of complementary food (children 6-23 months)^b^	48.3
Minimum dietary frequency of complementary food (children 6-23 months)^b^	73.6
Minimum acceptable diet (children 6-23 months)^b^	36.6
Age-appropriate feeding (children 0-23 months)	40.6

^a^ *N* = 309; ^b^ *N* = 1057

### Risk factors for stunting

Table 3 provides the results of the bivariate and multivariate analysis on the association of total stunting with child, maternal and household characteristics.

**Table 3:** Risk factors for total stunting in children aged 0-23 months

Factors		Stunted (%)	*N*	Unadjusted (bivariate)			Adjusted (multivariate)		
				OR	(95 % CI)	*P*	OR	(95 % CI)	*P*
Sex	Boys	30.9	689	1.29	(0.99 - 1.68)	0.06	1.45	(1.11 - 1.90)	0.007
	Girls	25.8	677	1.00			1.00		
Age of child	12 - 23 months	37.7	716	4.00	(2.72 - 5.87)	< 0.001	4.40	(2.97 - 6.53)	< 0.001
	6-11 months	22.6	341	1.91	(1.27 - 2.88)		1.97	(1.30 - 2.99)	
	0-5 months	13.3	309	1.00			1.00		
Breastfeeding within one hour of birth	No	30.1	438	1.14	(0.87 - 1.50)	0.34			
	Yes	27.6	928	1.00					
Age-appropriate feeding	No	31.3	812	1.39	(1.09 - 1.77)	0.008			
	Yes	24.2	554	1.00					
Mother’s age	≥ 40 years	28.3	92	1.26	(0.61 - 2.63)	0.79			
	30-39 years	29.3	516	1.38	(0.73 - 2.60)				
	20-29 years	28.1	704	1.29	(0.70 - 2.37)				
	< 20 years	24.1	54						
Mother’s education	No or incomplete primary	43.4	196	2.53	(1.77 - 3.62)	<0.001			
	Completed primary	31.0	229	1.48	(1.04 - 2.12)				
	Completed junior high	27.6	333	1.28	(0.96 - 1.72)				
	Completed senior high	23.0	608	1.00					
Number of household members	> 4	28.9	816	1.03	(0.83 - 1.29)	0.77			
	≤ 4	27.6	550	1.00					
Wealth quintile	Lowest	40.1	294	2.84	(1.84 - 4.40)	< 0.001	2.30	(1.43 - 3.68)	0.004
	Second	31.0	277	1.89	(1.33 - 2.70)		1.85	(1.26 - 2.72)	
	Third	27.1	273	1.56	(1.06 - 2.30)		1.68	(1.11 - 2.55)	
	Fourth	23.0	256	1.26	(0.83 - 1.91)		1.31	(0.85 - 2.04)	
	Highest	19.2	266	1.00			1.00		
Sanitation	Unimproved	35.3	532	1.71	(1.37 - 2.15)	<0.001	1.27	(0.99 - 1.63)	0.06
	Improved	24.0	834	1.00			1.00		
Safe disposal of child’s faeces	Unsafe	29.6	773	1.10	(0.87 - 1.40)	0.41			
	Safe	26.8	593	1.00					
Use of soap for hand washing	Not use soap	31.6	611	1.29	(1.00 - 1.67)	0.05			
	Use soap	25.8	755	1.00					
Water source	Unimproved	27.3	935	0.86	(0.67 - 1.10)	0.23			
	Improved	30.9	431	1.00					
Water treatment	Untreated	38.2	136	1.59	(1.08 - 2.34)	0.019	0.89	(0.49 - 1.61)	0.70
	Treated	27.3	1230	1.00			1.00		
Number of ANC visits of mother during pregnancy	< 4	40.7	91	1.70	(1.12 - 2.60)	0.013			
	≥ 4	27.5	1275	1.00					
Doctor/midwife provided ANC to mother during pregnancy	No	45.3	64	2.07	(1.29 - 3.33)	0.003			
	Yes	27.6	1302	1.00					
ANC in private or public health facility	No	45.8	48	2.12	(1.16 - 3.87)	0.014			
	Yes	27.8	1318	1.00					
Mother participates in decisions on HH food purchases	Yes	28.8	1211	1.21	(0.78 - 1.87)	0.39			
	No	25.2	155	1.00					
Mother participates in decisions on what food is cooked for HH	Yes	29.2	1223	1.50	(0.95 - 2.38)	0.08			
	No	21.7	143	1.00					
Mother participates in decisions on food given to child	Yes	28.5	1300	1.18	(0.61 - 2.27)	0.63			
	No	25.8	66	1.00					
Mother participates in decisions on seeking health care for child	Yes	28.7	1180	1.13	(0.79 - 1.61)	0.50			
	No	26.3	186	1.00					
Sanitation x water treatment							2.81	(1.32 - 5.97)	0.007

Using bivariate analysis, the prevalence of stunting was significantly higher among children aged 12-23 months (37.7 %) and children aged 6-11 months (22.6 %) than children aged 0-5 months (13.3 %). Children who were not given age-appropriate feeding were significantly more likely to be stunted than those who were fed appropriately (31.3 % vs. 24.2 %). The prevalence of stunting was higher among children whose mothers had not completed primary education (43.4 %) or completed primary education (31.0 %) compared with those who had completed high school (23.0 %). Children of poorer households, as measured by an asset-based wealth quintile, were significantly more likely to be stunted than wealthier households; the prevalence of stunting ranged from 19.2 % among the highest quintile to 40.1 % among the lowest wealth quintile. The stunting prevalence was significantly higher among children living in households that had an unimproved latrine compared with an improved latrine (35.3 % vs. 24.0 %); households that did not use soap for handwashing compared with those that did (31.6 % vs. 25.8 %); and households that drank untreated water compared with treated (38.2 % vs. 27.3 %). The stunting prevalence was also significantly lower among children of mothers who had good access to health care as indicated by an inadequate number of ANC visits, ANC care by a doctor or midwife, and ANC at a health facility.

Three variables were independently significant when entered into the multiple logistic model to control for potential confounding: child sex, child age, and household wealth quintile. Boys had a 45 % higher odds on being stunted than girls (AOR 1.45; 95 % CI 1.11-1.90). Children 12-23 months had an over four-fold odds on stunting than children aged 0-5 months (AOR 4.40; 95 % 2.97-6.53). Children from the lowest wealth quintile had more than twice the odds on being stunted than children from the highest wealth quintile (AOR 2.30; 95 % CI 1.43-3.68). In addition, there was an interaction between household sanitary facility and water treatment (*P* for interaction < 0.007): among children who lived in households that drank untreated water, the adjusted odds on stunting was over three times greater if the household used a unimproved latrine (AOR 3.47, 95 % CI 1.73-7.28, *P* < 0.001); however, among children who lived in households that drank treated water, the adjusted odds on stunting was not significantly higher if the household used an unimproved latrine (AOR 1.27, 95 % CI 0.99-1.63, *P* = 0.06).

We repeated the multivariate analysis for the dataset of children aged 0-35 months (*N* = 1937), excluding variables that were not available for children aged 24-35 months (IYCF practices and mother’s access to ANC during her most recent pregnancy). The same three variables were significant in the multiple logistic regression model (child sex, child age, wealth quintile) as well as the interaction between household sanitation facility and household water treatment (see Additional file 1: Table S1). Adjusting for other significant covariates, unimproved sanitation in household with untreated water was associated with more than twice the odds of stunting in their children (AOR 2.60, 95 % CI 1.37-4.93, *P* = 0.004), while unimproved sanitation in households that treated their water was associated with only 28 % more odds of stunting (AOR 1.28, 95 % CI 1.04-1.58, *P* = 0.02). In addition, the odds on being stunted were significantly greater among children whose mothers did not complete primary education compared with those who completed senior high school (AOR 1.67, 95 % CI 1.13-2.47).

### Risk factors for severe stunting

Table 4 provides the results for severe stunting children aged 0-23 months. Using bivariate analysis, the prevalence of severe stunting was significantly higher among children aged 12-23 months (8.8 %) than children aged 0-5 months (2.9 %). The prevalence of severe stunting was higher among children whose mothers had not completed primary education (19.4 %) or completed primary education (8.3 %) compared with those who completed high school (3.0 %). Children of poorer households were significantly more likely to be severely stunted than wealthier households; the prevalence ranged from 2.3 % among the highest quintile to 17.0 % among the lowest wealth quintile. The severe stunting prevalence was significantly higher among children living in households that had unimproved latrines compared with improved (10.5 % vs. 4.3 %); households that did not safely dispose of child faeces compared to those that did (8.9 % vs. 3.9 %); households that did not use soap for handwashing compared with those that did (10.0 % vs. 4.1 %); and households that drank untreated water compared with treated (14.7 % vs. 5.9 %). The severe stunting prevalence was significantly lower among children of mothers who had good access to health care as indicated by ANC care by a doctor or midwife and at a health facility. Children whose mothers participated in decisions on what food was cooked for the household had a higher prevalence of severe stunting than children of mothers who did not participate in these decisions (7.3 % vs. 2.1 %).

**Table 4:** Risk factors for severe stunting in children aged 0-23 months

Factors		Severely stunted (%)	*N*	Unadjusted (bivariate)			Adjusted (multivariate)		
				OR	(95 % CI)	*P*	OR	(95 % CI)	*P*
Sex	Boys	8.1	689	1.61	(0.97 - 2.67)	0.07	1.77	(1.07 - 2.93)	0.03
	Girls	5.3	677	1.00			1.00		
Age of child	12 - 23 months	8.8	716	3.34	(1.64 - 6.79)	0.002	3.27	(1.65 - 6.47)	< 0.001
	6-11 months	5.9	341	2.17	(0.91 - 5.15)		1.92	(0.82 - 4.48)	
	0-5 months	2.9	309	1.00			1.00		
Breastfeeding within one hour of birth	No	8.4	438	1.47	(0.94 - 2.29)	0.09			
	Yes	5.9	928	1.00					
Age-appropriate feeding	No	8.3	812	1.52	(0.96 - 2.41)	0.07			
	Yes	4.5	554	1.00					
Mother’s age	≥40 years	9.8	92	0.87	(0.27 - 2.88)	0.65			
	30-39 years	5.6	516	0.57	(0.21 - 1.52)				
	20-29 years	6.8	704	0.63	(0.25 - 1.59)				
	<20 years	11.1	54	1.00					
Mother’s education	No or incomplete primary	19.4	196	6.81	(3.59 - 12.91)	< 0.001			
	Completed primary	8.3	229	2.57	(1.38 - 4.77)				
	Completed junior high	5.1	333	1.77	(0.97 - 3.23)				
	Completed senior high	3.0	608	1.00					
Number of household members	> 4	7.7	816	1.39	(0.82 - 2.37)	0.22			
	≤ 4	5.3	550	1.00					
Wealth quintile	Lowest	17.0	294	8.62	(3.36 - 22.12)	< 0.001	8.48	(3.19 - 22.57)	< 0.001
	Second	6.1	277	2.80	(1.08 - 7.28)		2.93	(1.12 - 7.70)	
	Third	3.3	273	1.49	(0.53 - 4.22)		1.66	(0.59 - 4.69)	
	Fourth	3.9	256	1.77	(0.62 - 5.08)		1.89	(0.65 - 5.45)	
	Highest	2.3	266	1.00			1.00		
Sanitation	Unimproved	10.5	532	2.20	(1.40 - 3.47)	0.001			
	Improved	4.3	834	1.00					
Safe disposal of child’s faeces	Unsafe	8.9	773	1.92	(1.19 - 3.12)	0.008			
	Safe	3.9	593	1.00					
Use of soap for hand washing	Not use soap	10.0	611	2.04	(1.28 - 3.24)	0.003			
	Use soap	4.1	755	1.00					
Water source	Unimproved	6.6	935	1.04	(0.57 - 1.93)	0.89			
	Improved	7.0	431	1.00					
Water treatment	Untreated	14.7	136	2.19	(1.20 - 3.98)	0.011			
	Treated	5.9	1230	1.00					
Number of ANC visits of mother during pregnancy	< 4	12.1	91	1.35	(0.45 - 4.02)	0.59			
	≥ 4	6.4	1275	1.00					
Doctor/midwife provided ANC to mother during pregnancy	No	21.9	64	3.23	(1.51 - 6.92)	0.003			
	Yes	6.0	1302	1.00					
ANC in private or public health facility	No	25.0	48	4.04	(1.68 - 9.70)	0.002	2.58	(1.19 - 5.58)	0.02
	Yes	6.1	1318	1.00					
Mother participates in decisions on HH food purchases	Yes	6.8	1211	0.91	(0.47 - 1.76)	0.77			
	No	6.5	155	1.00					
Mother participates in decisions on what food is cooked for HH	Yes	7.3	1223	3.09	(1.13 - 8.44)	0.028			
	No	2.1	143	1.00					
Mother participates in decisions on food given to child	Yes	6.8	1300	1.11	(0.40 - 3.08)	0.85			
	No	6.1	66	1.00					
Mother participates in decisions on seeking health care for child	Yes	6.9	1180	1.22	(0.64 - 2.34)	0.55			
	No	5.9	186	1.00					

Four variables were independently significant when entered into the multiple logistic model to control for potential confounding: child sex, child age, mother’s education, and ANC care in a health facility. Mother’s education and wealth quintile were strongly associated (Pearson χ^2^ = 597, *P* < 0.001); when mother’s education was excluded from the multivariate analysis, wealth quintile was significantly associated with severe stunting and there was little change in the AOR of other variables. Given that it is easier to reverse low wealth status than poor education status, we retained wealth quintile in the multivariate analysis (Table 4). Boys had a 77 % higher odds on being severely stunted than girls (AOR 1.77; 95 % CI 1.07-2.93). Children 12-23 months had an over three-fold odds on stunting than children aged 0-5 months (OR 3.27; 95 % 1.65-6.47). Children in the lowest wealth quintile had more than eight-fold odds on being severely stunted than children in the highest wealth quintile (AOR 8.48; 95 % CI 3.19-22.57). Children whose mothers did not go to a health facility for ANC had over twice the odds on being severely stunted than children whose mothers did (AOR 2.58; 95 % CI 1.19-5.58).

We repeated the analysis for children aged 0-35 months (*N* = 1937), excluding variables that were not available for children aged 24-35 months (see Additional file 2: Table S2). Child sex, child age and wealth quintile were significantly associated with severe stunting in the multivariate analysis. In addition, the odds on being stunted were significantly greater among children whose mothers did not complete primary education compared with those who completed senior high school (AOR 3.30, 95 % CI 1.70-6.40).

## Discussion

Our analysis examined the factors associated with stunting in children aged 0-23 months from three districts in Indonesia. The prevalence of stunting in this population (28.4 %) was lower than the 2013 national survey data for Indonesia (32.9 %) [5] and similar to an 2004 survey in North Maluku Province in Indonesia (29.0 %) [12].

We found that the odds on being stunted in households that drank untreated water was over three times higher if the household used an unimproved latrine, while in households that drank treated water the odds on stunting were 27 % higher if the household used an unimproved latrine. Stunting was not associated with the household’s source of drinking water or method used to dispose of a child’s faeces. A significantly higher odds on stunting was observed among boys, older children and poorer households. For children aged 0-35 months, the odds on stunting were significantly greater among children whose mothers had completed less years of schooling; otherwise the results were similar to children aged 0-23 months.

The relationship between WASH and nutritional status has not been thoroughly investigated in Indonesia. Recent analyses of cross-sectional survey data on the determinants of child stunting in Indonesia [12, 13] did not consider or comment on associations with WASH variables. A study examining community determinants of child growth in an Indonesian tea plantation found that the density of latrines was significantly associated with improved nutritional status of children aged 6-18 months [14] but the analysis did not differentiate between improved and unimproved latrines. An evaluation of a sanitation programme in East Java found reductions in the prevalence of soil-transmitted helminths and improvements in height, weight and weight-for-height among children living in households that had an unimproved latrine at baseline; the effects were significant only for non-poor households, which were more likely to build latrines as a result of the programme [15].

Elsewhere, evidence of the association between sanitation and stunting in low and middle-income countries is growing. Analysis of data collected in eight countries across three continents showed that incremental improvements in sanitation were significantly associated with increases in child height [16]. Using data from 172 Demographic and Health Surveys (DHS) between 1986 and 2007, Fink et al. [17] examined within-country variations in stunting and sanitation and showed that the odds on stunting were lower in households that had access to improved sanitary facilities (OR: 0.73, 95 % CI 0.71–0.75). More recently, Spears [18] used DHS data from 65 countries to examine inter-country variations in stunting and sanitation, and estimated that variations in stunting explain 54 % of international variation in child height. In addition, a variety studies from individual countries involving cross-sectional survey analysis (India [19, 20]), longitudinal studies (Peru [21]) and operational research (Ethiopia [22]) indicate that improved sanitation is important for the linear growth of children.

The global evidence on the association between water source and stunting is less clear. Analysis of DHS data from 70 low and middle income countries [17] showed that an improved water source was associated with a lower odds on stunting (OR: 0.92, 95 % CI 0.89–0.94). Other studies have indicated that the protective effect of improved water is conditional on other WASH factors. Using data collected in the 1980s from eight countries in Africa, South Asia and South America, Esrey [16] found that the effect of water supply on child height was small and only positive among rural children when both improved sanitation was optimal and water services were available. More recently, Rah et al. [20] found that the protective effects of the mother or caregiver’s reported personal hygiene practices against stunting in India were stronger when they were accompanied by household access to piped water. In our study, the interaction between household treatment of drinking water and sanitation suggests that household treatment of water may provide some protective effect in households that have unimproved sanitation.

A meta-analysis of data from 14 cluster-randomized trials conducted in 10 low and middle income countries found a small benefit of WASH interventions (specifically solar disinfection of water, provision of soap, and improvement of water quality) on the height of in children under five years of age [23]. The analysis was constrained by the lack of studies of high methodological quality, particularly for sanitation.

Poor WASH facilities and behaviours may impact on child nutritional status by causing diarrhoea [24], intestinal worm infections [25], or environmental enteropathy [26]. These infections and conditions directly affect nutritional status through variations pathways including loss of appetite, loss of host tissues, maldigestion or malabsorption of nutrients, chronic immune activation, and other responses to infection that divert the use of nutrients and energy, such as fever.

We conducted a similar analysis to identify the determinants of severe stunting in children aged 0-23 months and 0-35 months. The direction of the bivariate associations between the WASH variables and severe stunting were similar to total stunting, however the associations did not remain significant in the multivariate model, possibly because the prevalence of severe stunting was relatively low in this population (6.7 % in children aged 0-23 months and 7.7 % in children aged 0-35 months).

Our findings of a higher odds on stunting and severe stunting among boys, older children, lower household wealth, and lower maternal education are consistent with findings in Indonesia [12, 13] and other low and middle-income countries [27–30].

There are several limitations in our analysis. First, we used cross-sectional data and so the analysis cannot provide evidence of a causal relationship between stunting and determinants. Randomized-controlled trials on the relation between sanitation and health is difficult to generate due to complexities in behaviour change of communities, variations in technological requirements, and the multitude of faecal contamination pathways [31, 32]. Second, data on personal and household practices was based on the mother’s recall, which may have been subject to bias. Third, the analysis did not include data on the mother’s nutritional status or the child’s birth weight or length, which are known predictors of stunting [13, 33]; birth weight data was available for only 823 children, while measurements of mother’s height and body mass index were not collected. However, we included indicators on household socio-economic status and health seeking behaviour that may partly explain mothers’ nutrition and health status. Fourth, children with chronic diseases were excluded and there could have been a relationship between the presence of some chronic diseases and stunting. However, these diseases are rarely diagnosed in children aged less than two years in Indonesia and so very few children would have been excluded. Fifth, the analysis did not include all determinants of household water quality, including safe water handling and storage prior to drinking [34]. Finally, the data were obtained from three districts, which are not necessarily representative of the country as a whole but all had a high prevalence and/or burden of stunting and represent different typologies in Indonesia.

Despite these limitations, the findings from this study have exposed associations between stunting and both household sanitation and water treatment in Indonesia that have previously not been reported. The findings are of immense concern to Indonesia because 20 % of all households in Indonesia, including 29 % of households in rural areas, defecate in the open, which amounts to over 51 million people [10], the second highest number in the world. They support the hypothesis that the variations between provinces in the proportion of households with no access to an improved latrine (Fig. 1) may partly explain the variations in stunting prevalence across Indonesia.

With stagnating levels of stunting a threat to the human and economic prosperity in Indonesia, these findings should be used to argue for greater prioritisation, capacity and investment in WASH interventions as part of the government’s efforts to reduce stunting in the first two years of life. Operational research is needed to generate further evidence on how to effectively converge and integrate WASH interventions with other stunting reduction interventions for greater impact on stunting prevalence and burden. The Sustainable Development Goals may present opportunities in this respect; increased collaboration between nutrition (Goal 2) and WASH (Goal 6) could be greatly encouraged by strong focus on high quality data and evidence of progress (Goal 17) [35, 36].

## Conclusions

In conclusion, our analysis shows that household sanitation and treatment of drinking water were strong predictors of stunting in a population of children aged 0-23 months in Indonesia. The findings add to the growing body of national and global evidence on the linkages between WASH and linear growth in early life, and indicate that policy and programme actions to address stunting should give greater attention to WASH interventions in Indonesia.

## Abbreviations

ANC, antenatal care; AOR, adjusted odds ratio; CI, confidence interval; DHS, Demographic and Health Survey; IYCF, infant and young child feeding; MYCNSIA, Maternal and Young Child Nutrition Security Initiative; OR, odds ratio; SUN, Scaling Up Nutrition; WASH, Water, sanitation and hygiene.

## Additional files

Additional file 1: Table S1. Risk factors for stunting in children age 0-35 months (*N* = 1937). (DOCX 21 kb) Additional file 2: Table S2. Risk factors for severe stunting in children age 0-35 months (*N* = 1937). (DOCX 21 kb)
