# Supplementary material for: Determinants of stunting in Indonesian children: evidence from a cross-sectional survey indicate a prominent role for the water, sanitation and hygiene sector in stunting reduction
Source: BMC Public Health. 2016 Jul 29;16:669. doi: 10.1186/s12889-016-3339-8 (PMC4966764; doi:10.1186/s12889-016-3339-8)
Supplement: Additional file 1: Table S1. — Risk factors for stunting in children age 0-35 months (N = 1937). (DOCX 21 kb) [file 12889_2016_3339_MOESM1_ESM.docx]

**Additional file 1: Table S1. Risk factors for stunting in children aged 0-35 months (N=1937)**

| **Factors** |  | **Unadjusted (bivariate)** | | | **Adjusted (multivariate)** | | |
| --- | --- | --- | --- | --- | --- | --- | --- |
|  |  | **OR** | **(95% CI)** | ***P*** | **OR** | **(95% CI)** | ***P*** |
| Sex | Boys | 1.20 | (0.98-1.48) | 0.073 | 1.34 | (1.08-1.66) | 0.009 |
|  | Girls | 1.00 |  |  | 1.00 |  |  |
| Age of child | 24-35 months | 4.90 | (3.36-7.15) | <0.001 | 5.58 | (3.77-8.25) | <0.001 |
|  | 12-23 months | 3.93 | (1.69-5.73) |  | 4.38 | (2.97-6.46) |  |
|  | 6-11 months | 1.87 | (1.24-2.81) |  | 1.93 | (1.28-2.90) |  |
|  | 0-5 months | 1.00 |  |  | 1.00 |  |  |
| Mother’s age | ≥40 years | 1.24 | (0.64-2.41) | 0.68 |  |  |  |
|  | 30-39 years | 1.35 | (0.76-2.40) |  |  |  |  |
|  | 20-29 years | 1.37 | (0.79-2.36) |  |  |  |  |
|  | <20 years | 1.00 |  |  |  |  |  |
| Mother's education | No or incomplete primary | 2.71 | (1.98-3.70) | <0.001 | 1.67 | (1.13-2.47) | 0.008 |
|  | Completed primary | 1.72 | (1.29-2.30) |  | 1.29 | (0.96-1.74) |  |
|  | Completed junior high | 1.53 | (1.20-1.95) |  | 1.39 | (1.08-1.80) |  |
|  | Completed senior high | 1.00 |  |  | 1.00 |  |  |
| Number of household members | >4 | 1.04 | (0.87-1.24) | 0.70 |  |  |  |
|  | ≤4 |  |  |  |  |  |  |
| Wealth quintile | Lowest | 3.13 | (2.14-4.56) | <0.001 | 2.01 | (1.28-3.17) | 0.002 |
|  | Second | 2.07 | (1.54-2.79) |  | 1.76 | (1.29-2.41) |  |
|  | Third | 1.97 | (1.45-2.68) |  | 1.92 | (1.37-2.70) |  |
|  | Fourth | 1.48 | (1.08-2.04) |  | 1.41 | (1.01-1.97) |  |
|  | Highest | 1.00 |  |  | 1.00 |  |  |
| Sanitation | Unimproved | 1.73 | (1.44-2.08) | <0.001 | 1.28 | (1.04-1.58) | 0.018 |
|  | Improved | 1.00 |  |  |  |  |  |
| Safe disposal of child’s faeces | Unsafe | 1.12 | (0.92-1.35) | 0.26 |  |  |  |
|  | Safe | 1.00 |  |  |  |  |  |
| Use of soap for hand washing | Not use soap | 1.31 | (1.06-1.62) | 0.013 |  |  |  |
|  | Use soap | 1.00 |  |  |  |  |  |
| Water source | Unimproved | 0.93 | (0.76-1.13) | 0.46 |  |  |  |
|  | Improved | 1.00 |  |  |  |  |  |
| Water treatment | Untreated | 1.55 | (1.11-2.16) | 0.010 |  |  |  |
|  | Treated | 1.00 |  |  |  |  |  |
| Mother participates in decisions on household food purchases | Yes | 1.13 | (0.81-1.58) | 0.49 |  |  |  |
|  | No | 1.00 |  |  |  |  |  |
| Mother participates in decisions on what food is cooked for HH | Yes | 1.43 | (0.99-2.08) | 0.057 |  |  |  |
|  | No | 1.00 |  |  |  |  |  |
| Mother participates in decisions on food given to child | Yes | 1.31 | (0.78-2.22) | 0.31 |  |  |  |
|  | No | 1.00 |  |  |  |  |  |
| Mother participates in decisions on seeking health care for child | Yes | 1.02 | (0.73-1.40) | 0.93 |  |  |  |
|  | No | 1.00 |  |  |  |  |  |
| Sanitation x water treatment |  |  |  |  | 2.03 | (1.06-3.87) | 0.032 |
