# Supplementary material for: Determinants of stunting in Indonesian children: evidence from a cross-sectional survey indicate a prominent role for the water, sanitation and hygiene sector in stunting reduction
Source: BMC Public Health. 2016 Jul 29;16:669. doi: 10.1186/s12889-016-3339-8 (PMC4966764; doi:10.1186/s12889-016-3339-8)
Supplement: Additional file 2: Table S2. — Risk factors for severe stunting in children age 0-35 months (N = 1937). (DOCX 21 kb) [file 12889_2016_3339_MOESM2_ESM.docx]

**Additional file 2: Table S2. Risk factors for severe stunting in children aged 0-35 months (N=1937)**

| **Factors** |  | **Unadjusted (bivariate)** | | | **Adjusted (multivariate)** | | |
| --- | --- | --- | --- | --- | --- | --- | --- |
|  |  | **OR** | **(95% CI)** | ***P*** | **OR** | **(95% CI)** | ***P*** |
| Sex | Boys | 1.66 | (1.19-2.31) | 0.003 | 1.86 | (1.31-2.66) | 0.001 |
|  | Girls | 1.00 |  |  |  |  |  |
| Age of child | 24-35 months | 4.14 | (1.99-8.60) | <0.001 | 4.38 | (2.13-9.00) | <0.001 |
|  | 12-23 months | 3.18 | (1.60-6.36) |  | 3.35 | (1.73-6.50) |  |
|  | 6-11 months | 2.04 | (0.87-4.80) |  | 1.96 | (0.85-4.53) |  |
|  | 0-5 months | 1.00 |  |  | 1.00 |  |  |
| Mother's age | ≥40 years | 0.65 | (0.22-1.94) | 0.88 |  |  |  |
|  | 30-39 years | 0.77 | (0.31-1.89) |  |  |  |  |
|  | 20-29 years | 0.75 | (0.31-1.82) |  |  |  |  |
|  | <20 years | 1.00 |  |  |  |  |  |
| Mother’s education | No or incomplete primary | 6.02 | (3.46-10.45) | <0.001 | 3.30 | (1.70-6.40) | 0.003 |
|  | Completed primary | 2.46 | (1.50-4.04) |  | 1.60 | (0.89-2.88) |  |
|  | Completed junior high | 2.05 | (1.26-3.33) |  | 1.97 | (1.00-2.94) |  |
|  | Completed senior high | 1.00 |  |  | 1.00 |  |  |
| Number of household members | >4 | 1.35 | (0.94-1.94) | 0.10 |  |  |  |
|  | ≤4 |  |  |  |  |  |  |
| Wealth quintile | Lowest | 8.67 | (4.09-18.37) | <0.001 | 4.91 | (1.93-12.51) | 0.014 |
|  | Second | 3.22 | (1.61-6.45) |  | 2.45 | (1.10-5.45) |  |
|  | Third | 2.45 | (1.19-5.02) |  | 2.25 | (1.06-4.80) |  |
|  | Fourth | 1.97 | (0.87-4.44) |  | 1.85 | (0.76-4.34) |  |
|  | Highest | 1.00 |  |  | 1.00 |  |  |
| Sanitation | Unimproved | 1.74 | (1.18-2.54) | 0.005 |  |  |  |
|  | Improved |  |  |  |  |  |  |
| Safe disposal of child’s faeces | Unsafe | 1.50 | (1.05-2.14) | 0.026 |  |  |  |
|  | Safe |  |  |  |  |  |  |
| Use of soap for hand washing | Not use soap | 1.74 | (1.21-2.51) | 0.003 |  |  |  |
|  | Use soap |  |  |  |  |  |  |
| Water source | Unimproved | 1.02 | (0.66-1.57) | 0.94 |  |  |  |
|  | Improved |  |  |  |  |  |  |
| Water treatment | Untreated | 1.82 | (1.13-2.92) | 0.014 |  |  |  |
|  | Treated |  |  |  |  |  |  |
| Mother participates in decisions on household food purchases | Yes | 1.17 | (0.65-2.11) | 0.60 |  |  |  |
|  | No |  |  |  |  |  |  |
| Mother participates in decisions on what food is cooked for HH | Yes | 1.69 | (0.85-3.32) | 0.13 |  |  |  |
|  | No |  |  |  |  |  |  |
| Mother participates in decisions on food given to child | Yes | 1.34 | (0.53-3.38) | 0.53 |  |  |  |
|  | No |  |  |  |  |  |  |
| Mother participates in decisions on seeking health care for child | Yes | 1.04 | (0.63-1.72) | 0.89 |  |  |  |
|  | No |  |  |  |  |  |  |
